# The Use of Fitness Influencers’ Websites by Young Adult Women: A Cross-Sectional Study

**DOI:** 10.3390/ijerph17176360

**Published:** 2020-09-01

**Authors:** Mariusz Duplaga

**Affiliations:** Department of Health Promotion and e-Health, Institute of Public Health, Faculty of Health Sciences, Jagiellonian University Medical College, Grzegórzecka Str. 20, 31-531 Krakow, Poland; mariusz.duplaga@uj.edu.pl

**Keywords:** fitness influencer, social media, health behaviors, physical activity, healthy nutrition, alcohol consumption, smoking, e-cigarettes, breast self-examination

## Abstract

The growth of the fitness industry observed in the last decade has been accompanied by the emergence of an occupation as a social media fitness influencer. The most popular are able to accumulate millions of followers. The marketing potential of fitness influencers is a subject of interest, not only for the fitness industry but also for other sectors offering products related to health, wellness, or healthy nutrition. However, the activities of fitness influencers related to the promotion of physical activity and healthy lifestyle converge with the aims of those promoting public health. The main objective of this study was to make an assessment of the determinants of regular access to fitness influencers’ sites (FIS) and their relationship with the health behaviors of young adult women. It was based on the data originating from an online survey on a representative sample of Polish women aged 18–35 years. Chi^2^ test, univariate, and multiply logistic regression models were used to determine the relationships between FIS and the variables related to the respondents’ characteristics of and their health behaviors. FIS were accessed by 29.3% of respondents (*n* = 1030) at least once a week. It was found that those living in cities with a population ranging from 20,000 to 100,000 were more likely to access FIS than those living in rural areas. Similarly, greater access was made by those in a high-income household rather than those with the lowest income, by those with inadequate rather than those with problematic health literacy and by those with high rather than low e-health literacy. The use of FIS was significantly associated with the consumptions of fruit and vegetables (OR, 95%CI: 2.77, 2.01–3.82), physical activity (1.74, 1.27–2.38), breast self-examination (1.54, 1.11–2.13), and also with the greater use of e-cigarettes (1.63, 1.09–2.43) and increased consumption of alcohol (1.37, 1.01–1.88). In conclusion, as access to Internet resources run by fitness influencers was a prevailing predictor of young adult Polish women’s health behaviors, FIS may play a potentially important role in promoting a healthy lifestyle in this population. However, it should be remembered that there are complex patterns of associations with specific behaviors, e.g., the use of e-cigarettes or alcohol consumption.

## 1. Introduction

The impressive growth of the fitness industry that has been observed in the last few years is driven by the demands of an increasing number of people demonstrating high awareness of their health and fitness. This interest in fitness is reflected by the growing population making use of fitness and health clubs and gyms. In 2019, it was estimated that 170 million people worldwide were members of health and fitness clubs [1]. Between 2009 and 2017, the number of fitness and health clubs worldwide increased from about 130,000 to 201,000 [2]. The Global Wellness Institute estimated that in 2017 the fitness and mind-body sector worldwide was worth nearly $600 billion and the healthy eating, nutrition, and weight loss sector about $700 billion [3]. The same Institute anticipates that the physical activity economy will be worth $1.1 trillion in 2023 [4].

The growth of the health and fitness industry has been accelerated by influencers active on the Internet. The role of social media influencer is one that has emerged in the last decade. Some of the social media influencers achieve the status of celebrities, however, they do not become famous because of any activities other than social media, but because they share their daily routines and images with a large audience [5]. This enables their followers to easily identify with them, which is more difficult to do if celebrity status having been for achievements in arts, journalism, or sport. In 2018, it was found that 20% of the world’s Internet users access social media sites to follow celebrities or celebrity news [6] and for Generation Z it reaches 26%. For them, social media is their principal source of information about brands and products. Interestingly, there are surveys which indicate that a significant number of people make purchases on the basis of influencers endorsement rather than that of the traditional celebrity or sporting icon [7].

The promotion of physical activity and healthy nutrition is frequently an element of the professional curriculum of fitness influencers. They gain popularity because they are able to satisfy the expectations of large groups of consumers who are intent on enhancing their health and wellness. Most influencers have the opportunity to attain at least the status of ‘micro-influencers’ with a limited number of loyal followers. Some manage to achieve real celebrity status in the area of fitness, healthy nutrition, or slimming, and they are able to generate considerable profits by promoting their own brands or the distribution of original products, e.g., exercise regimens, special diets, healthy food, sportswear, and even gadgets.

The influence exerted by fitness influencers on their followers may be an interesting topic in the context of the determinants influencing the health of modern societies. According to a periodic report prepared by the American College of Sports Medicine [8], four sectors may be identified in the fitness industry: commercial, based on the for-profit activities of companies; clinical, including medical fitness programs; community, mainly non-profit activities and corporate actions. The activities of the most popular fitness influencers that lead to independent Internet portals and offer fitness- and health-related products under their own brand, fall into the first category. Micro-influencers may also monetize their popularity, but they usually are hired or sponsored by large companies that offer products related to fitness, sport, or nutrition. The evolution from just a presence in social media and publishing own online content through the promotion of health and fitness-related products to professional business with own products and brands usually depends on the influencer’s popularity and the number of their followers [9]. There is also a category of influencers who do not treat social media as a source of income but regard it more like a communication channel to accompany their everyday coaching activities [9].

A commercial dimension of the services supporting health and fitness is the relatively new element of a complex network of health determinants. Already a classic model has been proposed by Dahlgren and Whitehead, which shows the relationship between the proximal and distal determinants of health [10]. Lifestyle and health-related behaviors are located in the central part of this model; a distal semicircle is formed by the socioeconomic and environmental determinants. Today, it is clear that the negative impacts of various industries should be included in the distal semicircle of determinants. The long-term supply of unhealthy product, e.g., highly processed food, accompanied by aggressive marketing pressures, results in unfavorable increase in the prevalence of noncommunicable diseases [11,12,13,14,15,16]. Such an association may be observed in both the highly developed and developing countries [17,18]. Some authors directly attribute the growing burden on society resulting from noncommunicable diseases to the unrestrained drive for profits by the international corporations [19].

The reports on consumption trends, especially among the younger generations, suggest that consumption of products with potential harmful health effects, such as highly processed food with high sugar or fat, is increasing [20,21,22,23]. It could also be seen that the marketing pressure of industries delivering such products prevails over countermeasures undertaken by public health systems, e.g., health education and other health promotion initiatives [24,25]. However, drastic, fiscal, or legal interventions, e.g., increased taxes on products with high sugar content or on alcohol beverages, seem to have a limited effect [26].

The fitness and healthy nutrition industry may be perceived as another sector, apart from the pharmaceutical industry fueling medicalization trends [27] and the industries supplying unhealthy products, such as highly-processed foods, tobacco products, or alcoholic beverages, which compete for modern consumers and are having a significant impact on their health. In terms of the priorities of public health and health promotion, shaping health awareness and inciting the demand for fitness and health-related products and services seems to be a beneficial development [28]. Paradoxically, only the efforts of the public sector, combined with the marketing messages from industries focusing on wellness, particularly the fitness and healthy nutrition sectors, may have sufficient power to establish the recommended lifestyle and beneficial health behaviors needed in modern societies [29,30,31]. It would appear that a beneficial effect of the fitness industry is at least partially related to the development of a specific healthy lifestyle culture that is frequently supported by social media [32,33,34].

The success stories of Internet-based fitness influencers raise the question as to what degree they may have an impact on the health behaviors of their followers. Do they only provide a surrogate of real-life activities or can they also influence the lifestyle of their online followers? A review of research on fitness influencers who are active on the Internet and social media shows that, to date, most researchers have been focused more on an assessment of the marketing benefits and their ability to modify the purchasing behaviors of their followers. Many fewer studies were aimed at understanding if the followers were likely to change their behaviors and adhere to a healthy lifestyle as a result of accessing the websites run by fitness influencers.

In 2018, a survey performed by GlobalWebIndex revealed that 41% of male and 30% of female Internet users in the UK and the USA admitted that influencers affect their purchasing decisions [6]. The influencers who provided information about food were followed by 43%, lifestyle and wellbeing was followed by 37%, and fitness by 30% of all followers of influencers [6]. According to the same report, younger groups of Internet users were more inspired by the posts generated by influencers (22% of generation Z, but only 6% of baby boomers). It would appear that the younger segments of the population are perceived as a priority target group for influencers.

In Poland, the fitness industry has great potential. According to the report prepared by EuropeActive and Deloitte, about 3 million Poles attended fitness and health clubs in 2018 [35]. This may be evidence of great interest and concern about health and fitness issues by a large segment of the Polish population. Furthermore, the fitness influencers most well-known to Polish users of the Internet are able to accumulate, depending on the type of social media, as many as 2–3 million followers [36,37,38].

To date, no study on the use of fitness influencers’ sites (FIS) by Polish women has been published. Consequently, it is not clear if the users of such websites have more beneficial healthy behavior and lifestyles. The study reported here is an analysis of the prevalence and factors associated with the use of FIS by young adult women in Poland. The association between the use of FIS and the health behaviors prevalent in this population were also assessed.

## 2. Materials and Methods

### 2.1. Survey

The survey was carried on a sample of adult women aged 18–35 (*n* = 1030) by means of the computer-assisted web-based interviewing (CAWI) technique in a two-week period in December 2018. The survey was conducted by a company experienced in conducting opinion polls in Poland, the PBS Company, on participants recruited from a certified Internet panel. The PBS Company adheres to the Polish Programme of Interviewer Quality Control [39] based on the Interviewer Quality Control Scheme (IQCS) [40]. The surveyed sample reflected the structure of the general female population aged 18–35 in Poland in terms of age, education (three categories), place of residence (4 categories), and the Nomenclature of Territorial Units for Statistics (NUTS) 1 region. The quota was established on the basis of the data reported by the Statistics Poland, the main statistical office in Poland [41]. The limitations of representativeness of the sample related to the sampling method and the recruitment were corrected using poststratification weights. These were generated with a random iterative method (RIM) for variables applied for the stratification of the samples and the main administrative units (voivodeships).

The questionnaire used in the survey consisted of 55 items. Standardized tools were used for the assessment of health literacy (HL) (a 16-item Health Literacy Survey Questionnaire (HLS-EU-Q16) [42]) and ehealth literacy (eHL) (an 8-item Polish version of the eHealth Literacy Scale (Pl-eHEALS) [43,44]). The questionnaire included also a set of the items asking about health-related use of the Internet, accessing sites conducted by Polish fitness influencers active on the Internet, health behaviors, a self-assessment of health status, opinions about vaccinations and homeopathy, the intake of dietary supplements, and the use of over-the-counter (OTC) or prescribed medication. The respondents were also asked questions related to sociodemographic characteristics. More details on the recruitment of the respondents were published earlier [44].

The study was accepted by the Bioethical Committee of Jagiellonian University in Krakow (No 122.6120.313.2016 of 24 November 2016). The questionnaire was completed anonymously by the survey participants after receiving information about the study and agreeing to participate.

### 2.2. Statistical Analysis

For the statistical analysis, IBM SPSS v.24 software (IBM Corp., Armonk, NY, USA) was used. Absolute and relative frequencies were calculated for the categorical variables and mean and standard deviation for the continuous variables. The chi^2^ test and univariate logistic regression models were developed to analyze the relationship between the use of fitness influencers’ sites (FIS) and the sociodemographic factors, HL, eHL, and the time spent using the Internet. For continuous variables, the differences between categories were assessed with either the t-Student test or the U Mann-Whitney test, depending on the distribution of the variable. The relationship between the use of fitness influencers’ sites (FIS) and health behaviors was assessed using multivariate logistic regression models after adjusting for HL, eHL, and sociodemographic variables. For the independent variables used in the logistic regression models, odds ratio (OR) and 95% confidence intervals (95%) were calculated. Where *p* < 0.10, the *p*-value was given to three decimal places, but where *p* > 0.10 to only two.

### 2.3. Variables

Bivariate logistic regression models were developed for the dependent variable reflecting the use of FIS at least once a week. Sociodemographic factors, having children, the total weekly duration of Internet use, the self-assessment of health status, the long-term use of diet supplements, the use of OTC and prescribed medication as well as the level of eHL and categorized HL were used as independent variables.

After adjusting for sociodemographic variables, HL and eHL multivariate logistic regression models were used for assessing the relationship between the use of FIS and health behaviors. Dependent variables were derived, after any necessary dichotomization, from items asking about smoking (yes vs. no), the use of e-cigarettes in the previous 30 days (at least once vs. no use), the consumption of alcohol in the previous 30 days (at least once vs. no consumption), physical activity (at least a few times in a week vs. not more than once a week), daily consumption of five portions of fruits and vegetable in the previous 30 days (at least a few time a week vs. not more than once a week), and breast self-examination (more often than once a year vs. once a year or less often).

The independent variables used in the multivariate logistic regression models, apart from the use of FIS, included the sociodemographic variables (age, level of education, place of residence, marital status, vocational activity, and net monthly income per inhabitant of the household), total weekly hours of Internet per week, and categorized HL and eHL. The total HL score was calculated only if there were at least 14 meaningful responses to the individual questions [42]. The response options “very difficult” and “difficult” were evaluated as “0” and “easy” and “very easy” as “1” [42,45]. Based on the total score, three categories of HL were established: “inadequate” for a score below 9, “problematic” for the range from 9 to 12, and “sufficient” for a score greater than 12 [42]. The eHL score was calculated as the sum of the scores for individual items, after assigning values from 1 to 5 to the response options (from “I decidedly do not agree” to “I decidedly agree”). The total eHL score ranged from 0 to 40.

## 3. Results

### 3.1. Characteristics of the Study Group

The mean age (standard deviation, SD) of the respondent was 26.09 (4.87) years. As for the level of education, 25.0% of respondents were graduates with a Bachelor’s or Master’s degree. There were 41.7% of inhabitants of rural areas and 27.0% of cities of at least 100,000 inhabitants. There were 56.0% singles in the study group, 40.0% married, and 4% were either widowed, separated, or divorced. The vocationally inactive were the largest group in the study (40.2%); 31.1% were employees in the public or private sector and 18.4% were college or university students. The characteristics of the study group are shown in Table 1.

There were 37.9% active smokers in the study group (Table 2), and 18.5% of respondents used e-cigarettes. Among respondents, 42.5% had consumed alcoholic beverages at least once in the previous 30 days and in the same period, 34.4% of respondents undertook some form of physical activity at least a few times a week. Fruits and vegetables were consumed five times a day every day, or nearly every day by 18.2% and a few times a week by 25.7% of the respondents in the previous 30 days. Finally, breast self-examination was performed more frequently than once yearly by only 29.3% of the respondents. Detailed presentation of the frequencies of health behaviors according to the categories of independent variables used in the multivariate logistic regression models has been included in Tables S1 and S2.

Weekly duration of the Internet not exceeding 3 h was stated by 42.3%, 31.7% indicated between 3 and 6 h, and 26.0% of the respondents spent more than 6 h using the Internet. The mean HL score (SD) was 11.87 (4.57) and the eHL score 29.52 (5.03). Only 16.0% of respondents assessed their health as unsatisfactory or satisfactory, 39.6% as good, and 44.5% as very good or perfect. As for the body mass index (BMI), 59.9% of the studied group were of normal weight, but 32.6% were overweight or obese, the remainder being underweight. In the study group, there were 34.5% persons admitting the use of diet supplements for prolonged periods, while 45.7% used OTC medication and 37.3% prescribed medication.

### 3.2. Factors Associated with the Use of Influencers’ Websites

Accessing FIS at least once a week was admitted by 29.3% of the respondents. There was a relationship between the accessing of FIS and the place of residence, net monthly income per member of the household, prolonged intake of diet supplements, and OTC medication, with both HL and eHL.

Women living in urban areas with a population ranging from 20,000 to <100,000 were 60% more likely to use FIS than those living in rural areas (OR, 95%CI: 1.59, 1.12–2.26). Furthermore, women living in households with the highest level of income were 1.5 times more likely to use FIS than those from households with the lowest income (OR, 95%CI: 1.54, 1.06–2.24). Interestingly, respondents using diet supplements or OTC medication for a prolonged period were less likely to access FIS (OR, 95%CI: 0.47, 0.35–0.62 and 0.75, 0.57–0.99, respectively).

An increase of 1 point in the eHL was associated with the increased likelihood of visiting FIS by 5% (OR, 95%CI: 1.05, 1.02–1.08). The use of FIS was confirmed by 35.2% of respondents having inadequate, by 24.2% of those with problematic HL and 29.1% of participants with sufficient HL. Univariate logistic regression revealed a significant difference between the respondents possessing inadequate and problematic HL (OR, 95%CI: 0.59, 0.37–0.94), but not between those with inadequate and sufficient HL (OR, 95%CI: 0.75, 0.52–1.08).

### 3.3. Determinants of Health Behaviours

The analysis based on multiple logistic regression showed a significant association between the use of FIS and all the analyzed health behaviors except smoking. Interestingly, the women who accessed FIS were more likely to use e-cigarettes (OR, 95%CI: 1.63, 1.09–2.43) and drink alcohol (OR, 95%CI: 1.37, 1.01–1.88) (Table 3). Furthermore, those visiting FIS more frequently consumed five portions of fruits and vegetables daily (OR, 95%CI: 2.77, 2.01–3.82), undertook physical activity (OR, 95%CI: 1.74, 1.27–2.38), and carried out breast self-examination (OR, 95%CI: 1.54, 1.11–2.13) (Table 4).

Smoking was associated with the level of education, vocational activity, and marital status. Respondents with the three higher levels of education were less likely to smoke than those with the level of education lower than upper secondary (OR, 95%CI: 6.71, 0.48–0.95; 0.13, 0.07–0.25; 0.23, 0.13–0.40 for comparison with upper secondary or post-secondary non-tertiary, Bachelor’s and Master’s degree level of education, respectively). University or college students were less likely to smoke than the employees in the public or private sector (OR, 95%CI: 0.36, 0.21–0.63). Finally, married, divorced, widowed, or separated respondents were less likely to smoke than singles (OR, 95%CI: 0.65, 0.46–0.92).

The use of e-cigarettes showed a statistically significant association, apart from the use of FIS, with age, education level, and the respondents’ vocational status. Older respondents were less prone to use e-cigarettes (OR, 95%CI: 0.91, 0.86–0.96). Persons with a Bachelor’s degree showed a lower likelihood of using e-cigarettes than those having the lowest level of education (OR, 95%CI: 0.28, 0.12–0.67). The comparison with respondents who held a Master’s degree did not show any statistical significance (OR, 95%CI: 0.48, 0.23–1.00). Vocationally inactive persons were less prone to use e-cigarettes than persons employed in the public or private sector (OR, 95%CI: 0.55, 0.34–0.90).

Alcohol consumption, apart from visiting FIS, was significantly related only to the respondent’s vocational status. Persons who were vocationally inactive less frequently consumed alcohol than employees (OR, 95%CI: 0.64, 0.45–0.92).

The consumption of the recommended amounts of fruit and vegetables was more frequent by those accessing FIS and those possessing higher HL and eHL (Table 5). Respondents with both problematic or sufficient level of HL were nearly twice as likely to consume five portions of fruits and vegetables daily than those with inadequate HL (OR, 95%CI: 1.89, 1.19–2.98, and 1.90, 1.28–2.81, respectively). The increase of the eHL score by 1 point was associated with a 4% increase in more frequently consuming the recommended daily amount of fruit and vegetables. Of interest, in the multivariate model, accessing FIS at least once a week was the only predictor of more intensive physical activity.

Breast self-examination was more frequently performed by the users of FIS and the respondents possessing a sufficient level of HL. In the latter group, the likelihood of more frequent self-examination was 1.8 higher than for those in the group with inadequate HL (OR, 95%CI: 1.80, 1.18–2.74) (Table 5).

## 4. Discussion

The most popular Polish Internet fitness influencers may have 1–3 million followers depending on the type of social media used. According to this study, nearly 30% of Polish women aged 18–35 years accessed FIS at least once weekly. For respondents living in cities with a population <20,000 or 20,000 to <100,000, the percentage of users was 35%. Interestingly, the lowest use of FIS was by the inhabitants in the largest cities (23%) and rural areas (26%). The difference between the inhabitants of rural areas and of cities with a population of 20,000 to <100,000 was statistically significant in the univariate logistic model.

The users of FIS also possessed significantly higher levels of eHL. Higher information skills and acceptance of the use of the Internet in a health-related context apparently encourages the users to access FIS. In the studied population, an increase of the eHL score by 1 point increased the likelihood of using FIS by 5%. The link between accessing social media sites on fitness and eHL was suggested earlier by Jong and Drummond [33].

The association between the visits to FIS and HL was not unequivocal. The highest usage of FIS, 35%, was by respondents possessing inadequate HL. The frequency of FIS usage among the respondents with problematic and sufficient HL was lower by 11 and 6 percent, respectively. However, logistic regression revealed a significant difference only for the comparison between inadequate and problematic HL. The lack of a difference between those with the lowest and the highest HL is perhaps surprising. However, due to the scarcity of studies focused on the characteristics of the followers of Internet fitness influencers, it is difficult to comment on the influence of HL on the use of FIS. It is possible that persons with higher HL are more inclined to engage in real-life health behaviors, e.g., they exercise in a gym regularly and, therefore, less often access FIS. They may be also repelled by the commercial flavor and marketing activities undertaken by many Internet fitness influencers.

Accessing FIS was lower for respondents who admitted a prolonged consumption of diet supplements or the use of OTC medication. There is not an obvious rationale to explain such a relationship. One can only hypothesize that the consumption of dietary supplements may be vicarious behavior replacing the access to FIS. It is also possible that some persons believe that taking supplements is an easier option than the demands of physical activity or diet regimens proposed by fitness influencers. However, some of the Internet fitness influencers are involved in marketing activities to supply supplements. Therefore, a negative relationship between accessing the FIS and the prolonged consumption of such products was not expected. The adverse association between the use of the FIS and the use of OTC medication is even more puzzling.

The multivariate analysis revealed that the use of FIS was associated with five of the six analyzed health behaviors, even after adjusting for HL and eHL and the sociodemographic factors. Unexpectedly, higher usage of FIS was associated with the more frequent use of e-cigarettes and alcohol consumption. Assuming that FIS users are more health-oriented, it is possible that e-cigarettes are regarded as being a less harmful option. For some users, it is probably also a means of reducing the use of tobacco. Regarding the consumption of alcohol, of those who admitted consuming alcoholic beverages at least once in the previous month, only 26% drank alcohol more frequently than once a week. Messages related to the consumption of alcoholic beverages currently appearing in the popular media are not consistent and so the higher consumption of alcohol by the users of FIS may paradoxically be evidence of the acceptance of a healthy lifestyle. In the past, medical associations suggested that alcohol consumption should not exceed one drink per day for women and two drinks per day for men [46,47]. An amended analysis of the available data and new studies tend to show that even low consumption of alcohol may be harmful and could lead to higher all-cause mortality and, specifically, of cancers [48]. Therefore, new dietary guidelines promote abstaining from alcohol [49]. However, this message does not transpire quickly to the social media and the Internet community.

Interestingly, from the independent variables included in the multivariate logistic regression model, only the use of FIS showed a significant relationship with the frequency of physical activity as a dependent variable. A similar effect was not observed for HL or eHL. It is not clear why none of the sociodemographic variables was related to physical activity. Furthermore, it may be rather disappointing that only 33% of young women were frequent participants in physical activities.

The phenomenon of the Internet and social media fitness influencers may be perceived as another manifestation of the e-health domain. The potential behind the use of the digital environment for the delivery of health-related interventions is well understood. Recent initiatives have shown that the public health community appreciates the benefits arising from digital health [50]. However, the results of the assessment of the effectiveness of ehealth interventions are not unequivocal [51]. According to the systematic review published by Rose et al., statistically significant changes of behaviors related to nutrition and physical activity by adolescents were observed for the interventions encompassing issues such as education, goal-setting, self-monitoring, and parental involvement [52]. The effect of technology-based programs on changing health behaviors among those aged 12–24 was assessed by Celik and Toruner [53]. They found that about 56% of studies reported significant changes to health behaviors that could result from the use of Internet-, computer- or mobile-based interventions. A systematic review focusing on new digital media interventions for sexual health promotion among young people revealed that only in 20% of studies in which the intention to use condoms for the prevention of HIV and other sexually transmitted infections were evaluated, a significant effect of the intervention was confirmed [54]. Another review reporting on the use of mobile applications in order to improve nutritional behavior showed the feasibility of such interventions [55]. It appears that the effectiveness of a digital intervention depends strongly on the behavior targeted and the type of technology applied [56,57,58,59].

Because of its popularity and an enormous number of users, social media were speedily adopted as one of the most significant channels for health communication. Social media are addressed both as the medium for the delivery of public health and health promotion interventions or as a predictor of potentially unfavorable consequences for those using them excessively. The use of social media for interventions targeting a change in the health behaviors in various groups of users has been the subject of many studies. The systematic review published in 2014 by Williams et al. was able to identify 22 studies assessing the effectiveness of social media interventions attempting to modify people’s diet and their physical activity [60]. A meta-analysis of the pooled results revealed no significant changes in physical activity, but a decrease in the consumption of dietary fat was identified as the result of social media interventions. The authors of a systematic review published in 2020 assessing the techniques used to achieve behavior change in social media interventions for promoting beneficial health behaviors in adults identified 71 relevant studies [61]. The techniques most frequently reported for such interventions included the provision of details on how to achieve the modified behavior, social support, self-monitoring of behavior, and information about possible health consequences. Another systematic review focused on interventions to encourage the cessation of smoking found that the interventions making use of social media were as effective as those based on individually designed interactive platforms [62]. The review of nutritional interventions targeting adolescents and young adults revealed that the majority of interventions that included a social media component achieved positive outcomes [63]. According to Hudnut-Beumler et al., social media were used for health promotion activities for the Hispanic populations addressing sexual health, healthy nutrition, and active living [64]. The growing use of social media in low and middle-income countries for health-related purposes was reported by Hagg et al. [65].

A systematic review carried out by Moorhead et al. based on 98 original studies showed that key benefits from the use of social media for health communication include: increased interactions, the greater availability of shared and tailored information; increased accessibility to and a widening access to health information, availability of peer, social, or emotional support; public health surveillance and the potential to influence health policy [66]. Giustini et al. indicated that the use of social media in public health initiatives and medicine may bring problems related to mental health, privacy, confidentiality, and the reliability of information [67]. Rounsefell et al. analyzed 26 original studies and found that social media engagement or exposure could be related to greater body dissatisfaction, dieting or restricting food, overeating, and choosing healthy foods among healthy young adults aged 18–30 [68]. Other authors pointed out that the advice given by so-called healthy food bloggers was not evidence-based and in the recipes analyzed by the authors, the fat content seemed to be increased to make up for the reduced sugar content [69]. Furthermore, some authors reported that messages posted on social media sites can promote health-damaging behavior, e.g., promoting anorexia [70]. Vannucci et al. carried out a systematic review with meta-analysis on the association between social media use and risky behaviors in adolescents [71]. They found a small to medium correlations between the use of social media and engagement in risky behaviors in general and related to substance abuse and risky sexual activities.

This study confirmed that, to some degree, the use of FIS may be associated with favorable health behaviors, at least in relation to physical activity, nutrition, and preventive activities. However, it is also related to the more frequent use of e-cigarettes and increased alcohol consumption.

Unfortunately, the impact of Internet fitness influencers on the health behaviors of their followers has been rarely addressed by researchers. Most available publications are focused on the commercial marketing role of fitness influencers who are active in social media. There are also many studies assessing the categories of health information sought by Internet users.

It seems that the emergence of Internet fitness influencers was a response to the significant interest in exercise and fitness already active in users of the Internet. The survey by the Pew Research Center in 2009 revealed that 52% of Internet users in the USA searched for information about exercise or fitness [72]. According to the Center, between 2002 and 2009, the percentage of adult Internet users visiting websites with information about exercise and fitness increased by 88% [72]. The report revealed that women searched for such information more frequently than men, as did younger rather than older people, those with higher rather than those with lower levels of education, and finally inhabitants of households with higher levels of income. In 2009, 56% of women, but only 48% of men, accessed fitness information online. Access was made to online fitness information by 61% of persons aged 18–29, by 57% of those aged 30–49, but only by 44% of the 50–64 years olds [72]. According to the 2015 Eurobarometer survey focused on digital health literacy, 74% of Europeans searching for health information online were seeking for information on aspects of lifestyle, including diet, nutrition, and physical activity (European Commission, 2014) [73].

In an Australia group of adolescents and young adults, greater interest in health and fitness was shown by the female members of the group [74]. The survey carried out on a sample of 15–29 years old respondents revealed that 30.8% accessed fitspiration websites and 23.5% sites providing plans for diet and fitness. The sociodemographic predictors of those who would access health and fitness-related social media content, apart from gender, included younger age, a higher level of education, and living outside a major city. Females were 3.5 times more likely than males to use such information, persons living outside a major city were twice as likely, and those in the youngest group (15–17 years old) were three times more likely than those in the oldest group (20–29 years old). Women were 13 times more likely to access websites offering diet and fitness plans than men. The authors of this study pointed out that the much higher proportion of women accessing health- and fitness-related social media is not surprising as the content is mainly aimed at them and is frequently driven by female celebrities and fitness models. The authors were concerned that up to 50% of young females were users of such online content because of it being frequently accompanied by objectifying messages. The finding of the Polish study reported here agree to a limited extent with those found in the Australian study. The use of FIS by the Polish female population 18–35 years old was not dependent on age and level of education. Nevertheless, respondents living outside major cities (but not from rural areas) were more likely to follow fitness influencers than inhabitants of the greatest cities.

Elavsky et al. carried out a study on the role of digital technologies for supporting health behaviors including diet, weight loss, and exercise or sport among young adults in the Czech Republic [75]. Of the 669 respondents aged 13–39, recruited from users of healthy lifestyle websites, 46.9% made daily, or almost daily, visits to websites on nutrition, similarly 16.9% for slimming advice and 37.2% for information on exercise and sport.

Fitness or nutrition is the most frequently searched topic online by teenagers. According to Wartella et al., 42% of teenagers aged 11–18 in a nationally representative sample in the USA searched for information on fitness and exercise and 36% on diet and nutrition [76]. Thirty-four percent of those who searched for health information (28% of all participants of the survey) admitted that they had changed their behavior as a result of the information obtained.

Dahl et al. undertook an analysis, based on structural equation modeling, of data received from a sample of 392 adult participants in a Country Wellness Rankings study carried out in one of the Midwestern states. They reported that access to primary care might increase the search for digital information, and this, in turn, may lead to changes in health behaviors and improved overall physical health [77].

Lee et al. analyzed the relationship between seeking online health information and the resulting health behaviors in a sample of nearly 2700 Hispanic residents in New York, NY, USA [78]. They defined online health information-seeking behavior as performing one of following three actions: participating in an online support group, using e-mail to communicate with physicians, and using the Internet to search for health or medical information. The analysis showed that health information seeking was significantly associated with the consumption of fruit, vegetables, and engaging in physical activity but not with the consumption of alcohol and adherence to medical regimens. The study also assessed HL; it was related to alcohol consumption and adherence to medical regimens. In our study, the use of FIS was significantly associated with all but one of the analyzed health behaviors. However, it should be noted that FIS users were more likely to use e-cigarettes and consume alcoholic beverages.

### Limitations

There are some limitations to this study that should be considered. It was a cross-sectional study, and consequently, the reasoning about the cause-effect relationship is not possible. Although the analysis of the association between the use of the FIS and health behaviors was adjusted for sociodemographic factors, HL and eHL, there are probably other predictors that were not included in the regression models. Furthermore, the questionnaire used in the study has not included items asking about the respondents’ self-assessment of any changes in their lifestyle or specific health behaviors that were the result of using FIS. The use of such questions could shed more light on a subjective feeling about the influence of fitness influencers on the lifestyle of participants, even if there was a risk of recall biases related to retrospective questions. The use of logistic regression models for the analysis of predictors of specific health behaviors required dichotomization of the relevant variables. This could result in the loss of some information, but this was a deliberate action to enable a more transparent presentation of the results. The impact of fitness influencers on the health behaviors of their followers involves several aspects, from health promotion, through marketing to e-health. It was not always possible to discuss all the relevant aspects despite the discussion becoming rather lengthy.

## 5. Conclusions

Health promotion is aimed at enabling people to efficiently take responsibility for their health and that of their family. It is a challenging task because the network of determinants influencing the health of modern societies is complex and not always transparent. It seems that the fitness influencers active on the Internet, especially those operating on social media, maybe allies of the public health professionals who frequently remain in conflict with the industries launching powerful marketing campaigns to sell products that may have a harmful impact on people’s health.

This study showed that there is a clear relationship between the use of the FIS and the health behaviors of young adult women. This group is particularly exposed to communications from fitness influencers and potentially there may be if the messages remain in line with the evidence given in public health and health promotion.

However, there is a risk that the activities of fitness influencers are dominated by commercial interests and attempts to generate profits from the sale of products that are worthless in terms of health benefits. The messages from the reported analysis are not always optimistic. The use of the FIS was associated with clearly positive behaviors such as greater physical activity, more beneficial nutrition patterns, or more active preventive behaviors but there are also some potentially harmful influences. The observed trend of the greater use of e-cigarettes and the consumption of alcohol beverages by FIS users requires further research because the messages generated by health care communities are not always explicit in regard to these issues.

## Figures and Tables

**Table 1:** The characteristics of the study group.

Variable	Response Categories	Number of Subjects % (*n*)
Education level	lower than upper secondary	34.1 (362)
	upper secondary or post-secondary non-tertiary	39.0 (401)
	bachelor’s degree	12.1 (124)
	masters’ degree or higher	13.8 (143)
Place of residence	rural	41.7 (430)
	urban < 20,000	9.7 (100)
	urban from 20,000 to <100,000	21.5 (222)
	urban from 100,000 to <500,000	16.2 (167)
	urban from 500,000	10.8 (111)
Marital status	single	56.0 (577)
	widowed, divorced or separated	4.0 (41)
	married	40.0 (412)
Vocational status	employee	31.1 (320)
	self-employed or farmer	10.3 (107)
	university or school student	18.4 (190)
	vocationally inactive including those on a disability pension	40.2 (414)
Net monthly income per household inhabitant	≤1000 PLN *	26.1 (268)
	1000–2000 PLN	34.6 (356)
	>2000 PLN	23.7 (245)
	refused to disclose	15.6 (161)
Children	no	39.6 (408)
	yes	60.4 (622)
Prolonged intake of diet supplements	no	55.5 (614)
	yes	34.5 (324)
Prolonged use of OTC	no	54.3 (524)
	yes	45.7 (441)
Long-term use of prescribed medication	no	62.7 (623)
	yes	37.3 (370)
Self-assessment of health status	not better than satisfactory	16.0 (164)
	good	39.6 (408)
	very good	31.6 (325)
	perfect	12.9 (132)
Body weight ^#^	underweight	7.5 (77)
	normal	59.9 (616)
	overweight	20.6 (212)
	obese	12.0 (124)
Weekly Internet use for non-professional purposes	not more than 3 h	42.3 (435)
	between 3 and 6 h	31.7 (326)
	more than 6 h	26.0 (268)
Health literacy	inadequate	20.9 (179)
	problematic	20.8 (178)
	sufficient	58.3 (499)
The use of fitness influencers’ sites	not at all or less often than once weekly	70.7 (729)
	at least once weekly	29.3 (301)

* PLN—current ISO4217 code for Polish zloty, ^#^ the categories of the body weight were established based on the level of the body mass index (BMI) calculated from the values of height and weight reported by the respondents: underweight—<18.5 kg/m^2^, normal weight—18.5–24.9 kg/m^2^, overweight—25.0–29.9 kg/m^2^, and obese–from 30.0 kg/m^2^.

**Table 2:** Health behaviors in the study group.

Health Behaviors	Categories of the Variable	% (*n*)
Smoking	yes	37.9 (390)
	no	62.1 (640)
E-cigarettes in the previous 30 days	at least once	18.5 (191)
	not used	81.5 (839)
Alcohol consumption in the previous 30 days	at least once	42.5 (437)
	no use	57.5 (593)
Physical activity in the previous 30 days	every day or nearly every day	11.8 (121)
	a few times a week	21.6 (222)
	not often than once a week	62.3 (642)
	not able to exercise	4.3 (45)
Consumption of five portions of fruits and vegetables daily in the previous 30 days	every day or nearly every day	18.2 (187)
	a few times a week	25.7 (264)
	once a week	13.6 (140)
	less often than once a week	42.6 (438)
Breast self-examination	more frequently than once yearly	29.3 (302)
	once yearly or less frequently	70.7 (727)

**Table 3:** Factors related to the use of fitness influencers’ sites.

Variable	Response Categories	The Use of Influencers’ Sites at least Once Weekly		*p* *	OR	95% CI	*p* ^&^
		No	Yes				
Education level	lower than upper secondary ^#^	70.7 (256)	29.3 (106)	0.33			0.33
	upper secondary or post-secondary non-tertiary	71.4 (287)	28.6 (115)		0.97	0.71–1.32	0.84
	bachelor’s degree	75.0 (93)	25.0 (31)		0.81	0.51–1.29	0.38
	masters’ degree or higher	65.0 (93)	35.0 (50)		1.31	0.87–1.97	0.20
Place of residence	rural ^#^	74.4 (319)	25.6 (110)	0.023			
	urban < 20,000	65.0 (65)	35.0 (35)		1.57	0.99–2.50	0.055
	urban from 20,000 to <100,000	64.4 (143)	35.6 (79)		1.59	1.12–2.26	0.009
	urban from 100,000 to <500,000	68.9 (115)	31.1 (52)		1.31	0.89–1.94	0.17
	urban from 500,000	77.5 (86)	22.5 (25)		0.83	0.51–1.36	0.46
Marital status	single	72.3 (417)	27.7 (160)	0.24			
	married, widowed, divorced or separated	68.9 (312)	31.1 (141)		1.18	0.9–1.54	0.24
Net monthly income per household member	≤1000 PLN ^%,#^	73.1 (196)	26.9 (72)	0.005			
	1000–2000 PLN	69.9 (249)	30.1 (107)		1.16	0.82–1.65	0.40
	>2000 PLN	63.7 (156)	36.3 (89)		1.54	1.06–2.24	0.025
	refusal	79.5 (128)	20.5 (33)		0.70	0.44–1.12	0.14
Vocational status	employee ^#^	70.6 (226)	29.4 (94)	0.45			
	self-employed or farmer	64.5 (69)	35.5 (38)		1.30	0.81–2.06	0.27
	university or school student	70.5 (134)	29.5 (56)		0.99	0.67–1.47	0.96
	vocationally inactive or on a disability pension	72.5 (300)	27.5 (114)		0.91	0.66–1.25	0.55
Children	no ^#^	72.7 (296)	27.3 (111)	0.26			
	yes	69.5 (432)	30.5 (190)		1.17	0.88–1.54	0.28
Prolonged intake of supplements	no ^#^	76.4 (469)	23.6 (145)	<0.001			
	yes	60.1 (194)	39.9 (129)		0.47	0.35–0.62	<0.001
Long-term use of prescribed medication	no ^#^	73.0 (455)	27.0 (168)	0.054			
	yes	67.3 (249)	32.7 (121)		0.76	0.57–1.001	0.051
Prolonged use of OTC ^@^	no ^#^	73.9 (387)	26.1 (137)				
	yes	67.8 (299)	32.2 (142)		0.75	0.57–0.99	0.041
Weekly Internet use for non-professional purposes	not more than 3 h ^#^	69.7 (304)	30.3 (132)	0.69			0.68
	between 3 and 6 h	70.6 (230)	29.4 (96)		0.96	0.7–1.32	0.82
	more than 6 h	72.8 (195)	27.2 (73)		0.86	0.61–1.21	0.39
Self-assessment of health status	not better than satisfactory ^#^	77.1 (37)	22.9 (11)	0.084			0.087
	good	73.3 (85)	26.7 (31)		1.23	0.56–2.7	0.61
	very good	73.8 (301)	26.2 (107)		1.19	0.59–2.42	0.62
	perfect	66.7 (305)	33.3 (152)		1.67	0.83–3.37	0.15
Body weight	underweight ^#^	79.2 (61)	20.8 (16)	0.22			
	normal	69.3 (427)	30.7 (189)		1.70	0.95–3.03	0.073
	overweight	69.7 (147)	30.3 (64)		1.68	0.90–3.13	0.11
	obese	74.8 (92)	25.2 (31)		1.31	0.66–2.59	0.45
Health literacy	inadequate ^#^	64.8 (116)	35.2 (63)	0.071			0.078
	problematic	75.8 (135)	24.2 (43)		0.59	0.37–0.94	0.025
	sufficient	70.9 (354)	29.1 (145)		0.75	0.52–1.08	0.13
Age ^$^		26.22 (4.96)	25.78 (4.64)	0.12	0.98	0.95–1.01	0.18
eHealth literacy ^$^		29.19 (5.07)	30.31 (4.85)	<0.001	1.05	1.02–1.08	0.001

* *p* value for chi^2^ test in the case of categorical variables, for the t-Student test in case of age, and U Mann-Whitney test in case of HL and eHL scores; ^&^ *p* value for univariate logistic regression; ^$^ for age, HL and eHL scores–mean (standard deviation) was provided depending on the category of the opinion about vaccinations; ^#^ reference category of the independent variable in the logistic regression model; ^%^ PLN—current ISO4217 code for Polish zloty, ^@^ OTC—over-the-counter used in relation to the medication sold without a prescription.

**Table 4:** Multivariate logistic regression model for smoking, the use of e-cigarettes, and alcohol consumption as dependent variables.

Independent Variables	Categories of an Independent Variable	Smoking			e-Cigarettes			Alcohol Consumption		
		OR	95% CI	*p* ^&^	OR	95% CI	*p* ^&^	OR	95% CI	*p* ^&^
The use of influencers’ websites	less often ^#^ vs. more often than once weekly	1.21	0.87–1.69	0.25	1.63	1.09–2.43	0.016	1.37	1.01–1.88	0.046
Health literacy	inadequate ^#^									
	problematic	1.48	0.94–2.34	0.092	1.28	0.75–2.17	0.37	1.21	0.78–1.86	0.39
	sufficient	0.94	0.63–1.4	0.75	0.63	0.39–1.02	0.059	0.79	0.54–1.14	0.21
eHealth literacy		1.01	0.98–1.05	0.40	1.02	0.98–1.06	0.43	1.00	0.97–1.03	0.88
Age		0.99	0.96–1.04	0.82	0.91	0.86–0.96	0.001	0.98	0.94–1.02	0.39
Place of residence	rural ^#^									
	urban < 20,000	1.10	0.64–1.91	0.73	0.77	0.36–1.65	0.50	1.02	0.63–1.77	0.83
	urban from 20,000 to <100,000	1.12	0.75–1.68	0.57	1.51	0.92–2.5	0.11	1.10	0.74–1.62	0.65
	urban from 100,000 to <500,000	1.59	0.99–2.52	0.052	1.19	0.66–2.14	0.57	1.21	0.78–1.86	0.39
	urban from 500,000	1.07	0.61–1.89	0.82	1.37	0.69–2.68	0.37	1.51	0.90–2.51	0.12
Net income per household member	≤1000 PLN ^%,#^									
	1000–2000 PLN	1.25	0.85–1.83	0.26	1.01	0.61–1.66	0.98	0.98	0.67–1.42	0.89
	>2000 PLN	1.14	0.74–1.76	0.55	0.99	0.57–1.75	0.99	1.10	0.73–1.67	0.65
	refused to disclose	0.99	0.61–1.62	0.97	1.74	0.98–3.1	0.058	1.41	0.89–2.23	0.14
Education level	lower than upper secondary ^#^									
	upper secondary or post-secondary non-tertiary	0.67	0.48–0.95	0.023	0.89	0.58–1.35	0.57	1.10	0.78–1.56	0.58
	bachelor’s degree	0.13	0.07–0.25	<0.001	0.28	0.12–0.67	0.004	1.62	0.97–2.70	0.063
	masters’ degree or higher	0.23	0.13–0.4	<0.001	0.48	0.23–1.00	0.050	1.19	0.72–1.97	0.49
Vocational status	employee ^#^									
	self-employed or farmer	1.01	0.59–1.74	0.96	0.98	0.5–1.92	0.94	0.68	0.41–1.13	0.14
	university or school student	0.36	0.21–0.63	<0.001	0.82	0.44–1.51	0.52	1.09	0.66–1.79	0.75
	vocationally inactive	0.99	0.68–1.44	0.95	0.55	0.34–0.90	0.018	0.64	0.45–0.92	0.017
Marital status	singles ^#^ vs. other	0.65	0.46–0.92	0.015	1.26	0.80–1.99	0.32	0.87	0.63–1.21	0.40

^&^*p* value for multivariate logistic regression; ^#^ reference category of the independent variable in the logistic regression model; ^%^ PLN—current ISO4217 code for Polish zloty.

**Table 5:** Multivariate logistic regression model for the consumption of fruits and vegetables, physical activity, and breast self-examination as dependent variables.

Independent Variables	Categories of an Independent Variable	Consumption of Fruits and Vegetables			Physical Activity			Breast Self-Examination		
		OR	95% CI	*p* ^&^	OR	95% CI	*p* ^&^	OR	95% CI	*p* ^&^
The use of influencers’ websites	less often ^#^ vs. more often than once weekly	2.77	2.01–3.82	<0.001	1.74	1.27–2.38	0.001	1.54	1.11–2.13	0.010
Health literacy	inadequate ^#^									
	problematic	1.89	1.19–2.98	0.007	1.18	0.74–1.88	0.48	1.26	0.77–2.06	0.37
	sufficient	1.90	1.28–2.82	0.001	1.45	0.98–2.16	0.066	1.80	1.18–2.74	0.006
eHealth literacy		1.04	1.003–1.07	0.031	1.02	0.99–1.06	0.14	1.02	0.98–1.05	0.39
Age		0.99	0.95–1.03	0.62	0.99	0.95–1.03	0.59	1.03	0.98–1.07	0.24
Place of residence	rural ^#^									
	urban < 20,000	1.11	0.65–1.88	0.71	0.93	0.54–1.61	0.80	0.77	0.44–1.36	0.37
	urban from 20,000 to <100,000	0.77	0.52–1.14	0.19	1.17	0.78–1.74	0.45	0.88	0.58–1.33	0.55
	urban from 100,000 to <500,000	0.71	0.45–1.12	0.14	1.21	0.77–1.89	0.40	1.15	0.73–1.8	0.56
	urban from 500,000	1.07	0.63–1.81	0.80	1.07	0.63–1.82	0.81	0.91	0.52–1.58	0.73
Net income per household member	≤1000 PLN ^%,#^									
	1000–2000 PLN	0.90	0.61–1.31	0.58	1.40	0.95–2.07	0.088	1.42	0.95–2.12	0.086
	>2000 PLN	1.37	0.90–2.10	0.14	1.19	0.77–1.84	0.44	1.20	0.77–1.88	0.42
	refused to disclose	1.34	0.83–2.14	0.23	1.11	0.68–1.81	0.67	1.07	0.64–1.78	0.79
Education level	lower than upper secondary^#^									
	upper secondary or post-secondary non-tertiary	1.08	0.76–1.54	0.65	1.01	0.71–1.45	0.95	1.36	0.94–1.97	0.11
	bachelor’s degree	1.05	0.62–1.78	0.85	0.96	0.56–1.64	0.89	1.52	0.89–2.59	0.13
	masters’ degree or higher	1.40	0.84–2.33	0.20	1.64	0.98–2.73	0.058	1.22	0.71–2.07	0.47
Vocational status	employee ^#^									
	self-employed or farmer	1.17	0.7–1.95	0.55	0.87	0.51–1.49	0.61	1.08	0.64–1.83	0.78
	university or school student	0.54	0.32–0.91	0.02	1.32	0.78–2.21	0.30	0.95	0.55–1.65	0.87
	vocationally inactive	0.87	0.6–1.25	0.45	1.00	0.69–1.46	0.98	0.98	0.67–1.44	0.92
Marital status	singles ^#^ vs. other	1.17	0.84–1.63	0.36	0.94	0.67–1.33	0.73	0.95	0.67–1.35	0.77

^&^*p* value for multivariate logistic regression; ^#^ reference category of the independent variable in the logistic regression model; ^%^ PLN—current ISO4217 code for Polish zloty.

## Supplementary Materials

The following are available online at https://www.mdpi.com/1660-4601/17/17/6360/s1, Table S1: The frequencies of health behaviors in the categories of independent variables used in the multivariate logistic regression models. Part I., Table S2: The frequencies of health behaviors in the categories of independent variables used in the multivariate logistic regression models. Part II.
