# Supplementary material for: The Use of Fitness Influencers’ Websites by Young Adult Women: A Cross-Sectional Study
Source: Int J Environ Res Public Health. 2020 Sep 1;17(17):6360. doi: 10.3390/ijerph17176360 (PMC7503997; doi:10.3390/ijerph17176360)
Supplement: Supplementary file 1 [file ijerph-17-06360-s001.pdf]

Table S1. The frequencies of health behaviours in the categories of independent variables used in the multivariate logistic regression models. Part I.

Variable	Categories of the Variable	Smoking		E-cigarettes in the Previous 30 days		Alcohol Consumption in the Previous 30 Days		Breast Self-Examination	
		yes	no	at least once	not used	at least once	no use	more frequently than once yearly	once yearly or less frequently
The use of influencers' websites	less often than once weekly	37,2 (271)	62,8 (458)	16,3 (119)	83,7 (609)	41 (299)	59 (430)	27,3 (199)	72,7 (529)
	more often than once weekly	39,5 (119)	60,5 (182)	23,6 (71)	76,4 (230)	46,2 (139)	53,8 (162)	34,2 (103)	65,8 (198)
Health literacy	inadequate	35.2 (63)	64.8 (116)	20.2 (36)	79.8 (142)	46.4 (83)	53.6 (96)	23 (41)	77 (137)
	problematic	43.3 (77)	56.7 (101)	23.6 (42)	76.4 (136)	51.1 (91)	48.9 (87)	26.6 (47)	73.4 (130)
	sufficient	36.5 (182)	63.5 (317)	14.4 (72)	85.6 (427)	39.5 (197)	60.5 (302)	34.9 (174)	65.1 (324)
Place of residence	rural	37.7 (162)	62.3 (268)	17 (73)	83 (357)	37.7 (162)	62.3 (268)	28.6 (123)	71.4 (307)
	urban <20.000	34 (34)	66 (66)	11 (11)	89 (89)	39 (39)	61 (61)	30 (30)	70 (70)
	urban from 20.000 to <100.000	42.1 (93)	57.9 (128)	22.6 (50)	77.4 (171)	42.1 (93)	57.9 (128)	28.4 (63)	71.6 (159)
	urban from 100.000 to <500.000	40.1 (67)	59.9 (100)	21 (35)	79 (132)	48.5 (81)	51.5 (86)	34.1 (57)	65.9 (110)
	urban from 500.000	30.6 (34)	69.4 (77)	19.6 (22)	80.4 (90)	55.9 (62)	44.1 (49)	26.4 (29)	73.6 (81)
Net income per household member	≤1,000 PLN	37.2 (100)	62.8 (169)	16.8 (45)	83.2 (223)	38.1 (102)	61.9 (166)	25.7 (69)	74.3 (199)
	1.000-2.000 PLN	40.2 (143)	59.8 (213)	18 (64)	82 (292)	40.7 (145)	59.3 (211)	33.4 (119)	66.6 (237)
	>2.000 PLN	38.1 (93)	61.9 (151)	18 (44)	82 (201)	45.5 (111)	54.5 (133)	30.3 (74)	69.7 (170)
	refused to disclose	33.5 (54)	66.5 (107)	23.6 (38)	76.4 (123)	48.4 (78)	51.6 (83)	25 (40)	75 (120)
Education level	lower than upper secondary	49 (177)	51 (185)	23 (83)	77 (278)	37 (135)	62.7 (227)	24 (86)	76 (275)
	upper secondary or post-secondary non-tertiary	40 (161)	60 (241)	20.4 (82)	79.6 (320)	41.4 (166)	58.6 (235)	31 (124)	69 (276)
	bachelor's degree	14.5 (18)	85.5 (106)	7.3 (9)	92.7 (115)	53.2 (66)	46.8 (58)	35.5 (44)	64.5 (80)
	masters' degree or higher	23.9 (34)	76.1 (108)	11.9 (17)	88.1 (126)	49 (70)	51 (73)	33.1 (47)	66.9 (95)
Vocational status	employee	34.7 (111)	65.3 (209)	15.3 (49)	84.7 (271)	48.1 (154)	51.9 (166)	32 (102)	68 (217)
	self-employed or farmer	34.6 (37)	65.4 (70)	20.6 (22)	79.4 (85)	38.3 (41)	61.7 (66)	34 (36)	66 (70)

	university or school student	32.1 (61)	67.9 (129)	31.2 (59)	68.8 (130)	52.4 (99)	47.6 (90)	23.8 (45)	76.2 (144)
	vocationally inactive	43.6 (180)	56.4 (233)	14.5 (60)	85.5 (353)	34.6 (143)	65.4 (270)	28.5 (118)	71.5 (296)
Marital status	singles	40.9 (236)	59.1 (341)	21.1 (122)	78.9 (455)	47.1 (272)	52.9 (305)	27.8 (160)	72.2 (416)
	other	34 (154)	66 (299)	15.2 (69)	84.8 (384)	36.5 (165)	63.5 (287)	31.3 (142)	68.7 (311)

Table S2. The frequencies of health behaviours in the categories of independent variables used in the multivariate logistic regression models. Part II.

Variable	Categories of the Variable	Physical Activity in the Previous 30 Days				Consumption of Five Portions of Fruits and Vegetables Daily in the Previous 30 Days			
		yes	no	at least once	not used	at least once	no use	more frequently than once yearly	once yearly or less frequently
The use of influencers' websites	less often than once weekly	11,8 (86)	17,7 (129)	65,3 (477)	5,2 (38)	14,5 (106)	23,3 (170)	12,2 (89)	49,9 (364)
	more often than once weekly	11,9 (36)	31 (94)	54,8 (166)	2,3 (7)	26,9 (81)	31,2 (94)	16,9 (51)	24,9 (75)
Health literacy	inadequate	8,9 (16)	20,1 (36)	63,7 (114)	7,3 (13)	13,4 (24)	19,6 (35)	20,7 (37)	46,4 (83)
	problematic	9,6 (17)	22,6 (40)	65 (115)	2,8 (5)	20,8 (37)	23 (41)	9,6 (17)	46,6 (83)
	sufficient	14,2 (71)	23 (115)	58,5 (292)	4,2 (21)	19,1 (95)	28,5 (142)	11,8 (59)	40,6 (202)
Place of residence	rural	11 (47)	18,4 (79)	66,2 (284)	4,4 (19)	18,1 (78)	26,7 (115)	14 (60)	41,2 (177)
	urban <20.000	8,9 (9)	23,8 (24)	62,4 (63)	5 (5)	19 (19)	31 (31)	14 (14)	36 (36)
	urban from 20.000 to <100.000	12,7 (28)	24,9 (55)	58,4 (129)	4,1 (9)	16,7 (37)	25,7 (57)	14 (31)	43,7 (97)
	urban from 100.000 to <500.000	12 (20)	24,6 (41)	59,3 (99)	4,2 (7)	20,4 (34)	19,2 (32)	13,2 (22)	47,3 (79)
	urban from 500.000	14,4 (16)	21,6 (24)	59,5 (66)	4,5 (5)	17,1 (19)	26,1 (29)	11,7 (13)	45 (50)
Net income per household member	≤1,000 PLN	11,5 (31)	16,3 (44)	65,9 (178)	6,3 (17)	17,9 (48)	22 (59)	14,6 (39)	45,5 (122)
	1.000-2.000 PLN	12,1 (43)	24,4 (87)	60,4 (215)	3,1 (11)	16,3 (58)	25,4 (90)	13,5 (48)	44,8 (159)
	>2.000 PLN	11 (27)	24,1 (59)	62,4 (153)	2,4 (6)	21,7 (53)	29,1 (71)	14,3 (35)	34,8 (85)
	refused to disclose	12,5 (20)	20,6 (33)	60 (96)	6,9 (11)	17,4 (28)	27,3 (44)	10,6 (17)	44,7 (72)
Education level	lower than upper secondary	12 (43)	20 (71)	63 (228)	5 (19)	16 (59)	25 (90)	15 (55)	43 (157)
	upper secondary or post-secondary non-tertiary	11,4 (46)	20,4 (82)	63,9 (257)	4,2 (17)	17,7 (71)	26,6 (107)	12,2 (49)	43,5 (175)
	bachelor's degree	9,6 (12)	26,4 (33)	62,4 (78)	1,6 (2)	19,4 (24)	20,2 (25)	17,7 (22)	42,7 (53)
	masters' degree or higher	14,7 (21)	25,9 (37)	55,2 (79)	4,2 (6)	23,8 (34)	29,4 (42)	9,1 (13)	37,8 (54)
Vocational status	employee	12,2 (39)	22,5 (72)	60,6 (194)	4,7 (15)	20,9 (67)	26,6 (85)	13,8 (44)	38,8 (124)
	self-employed or farmer	10,4 (11)	21,7 (23)	66 (70)	1,9 (2)	22,4 (24)	29,9 (32)	15 (16)	32,7 (35)

	university or school student	9,9 (19)	29,3 (56)	58,1 (111)	2,6 (5)	15,3 (29)	18,9 (36)	13,2 (25)	52,6 (100)
	vocationally inactive	12,3 (51)	17,4 (72)	64,6 (267)	5,6 (23)	16,4 (68)	27,1 (112)	13,3 (55)	43,2 (179)
Marital status	singles	12,7 (73)	22,9 (132)	60,3 (348)	4,2 (24)	17,7 (102)	22,7 (131)	13 (75)	46,5 (268)
	other	10,6 (48)	20,1 (91)	64,7 (293)	4,6 (21)	18,9 (86)	29,3 (133)	14,3 (65)	37,4 (170)
